# Association between tobacco smoke exposure and depression: the NHANES 2005–2018 and Mendelian randomization study

**DOI:** 10.1186/s13690-024-01322-4

**Published:** 2024-07-03

**Authors:** Yikun Guo, Jun Yan

**Affiliations:** 1 Department of Respiratory, Dongzhimen Hospital, Beijing University of Chinese Medicine, Dongcheng District, Beijing, 100700 China; 2 Beijing University of Chinese medicine, Chaoyang District, Beijing, 100029 China

**Keywords:** Tobacco smoke exposure, Smoking, Cotinine, NHANES, Mendelian randomization

## Abstract

**Objective:**

The relationship between tobacco smoke exposure (TSE) and depression is controversial. This study combined observational research and Mendelian randomization (MR) to explore the relationship of depression with both smoking status and cotinine levels.

**Method:**

We collected relevant data from the National Health and Nutrition Examination Survey (NHANES) database from 2005 to 2018, and used weighted multifactorial logistic regression modelling to assess the correlation between TSE and depression, and assessed the causal relationship of depression with both smoking status and cotinine levels by MR.

**Result:**

Current smokers had the highest risk of depression (OR 1.94; *P* < 0.01); there was a positive trend for correlation between daily smoking and depression (OR 1.66; P for trend < 0.01). Serum ketamine levels above 3.00 ng/ml had a higher risk of depression (OR 2.13; *P* < 0.001). MR results showed that current smoking (OR = 4.66; *P* < 0.001) and previous smoking (OR 2.09; *P* < 0.01) were risk factors for the onset of depression, and that there was no causal association between cotinine levels and depression.

**Conclusion:**

Smoking is significantly associated with depression and plays a potential causal role in the development of depression. Cotinine was significantly associated with depression, however MR results showed no causal relationship between cotinine and depression.

**Supplementary Information:**

The online version contains supplementary material available at 10.1186/s13690-024-01322-4.

**Table:** 

Text box 1. Contributions to the literature
There is limited evidence on contributions of healthcare systems and public health policies in tobacco smoke exposure and depression, and there is controversy over the relationship, especially the causal relationship is not yet clear.There are many factors that can cause depression, but exposure to tobacco smoke is a very important factor in leading to depression, as evidenced by smoking status, smoking cessation duration, smoking intensity, and serum cotinine levels.
Combining observational studies with Mendelian randomization analysis can provide a more accurate understanding of the association between tobacco smoke exposure and depression, informing the formulation of public health policies.

## Introduction

Depression is a significant public health issue affecting approximately 350 million people worldwide [1, 2]. It is characterized by persistent symptoms of low mood and is associated with decreased social behavior and quality of life. Depression can also lead to nonfatal health losses and is one of the leading causes of disability and death globally [3, 4]. Research indicates that depression affects at least one-fifth of the global population at some point in their lives, resulting in socioeconomic losses and health care resource depletion [5].

The development of depression is a multifaceted process that is affected by genetic, psychological, environmental, and biological factors [6]. Although there is mounting evidence that smoking may contribute to psychological issues, the relationship between smoking and the risk of developing depression has been a topic of debate [7]. A study utilizing Mendelian randomization (MR), a causal inference method that is based on genetic variations, demonstrated that smoking is linked to a greater risk of developing depression and even schizophrenia [8]. . Research has demonstrated that quitting smoking can enhance mental health, even in individuals with mental illness. Additionally, maintaining abstinence for more than a decade has been linked to a decreased risk of major depression [9, 10]. In contrast, a systematic review of the attitudes of mental health professionals towards smoking and smoking cessation in individuals with mental illness (comprising 38 small studies and 16,369 participants) revealed that smoking alleviated several mental health issues, such as depression, anxiety, and stress. Additionally, quitting smoking intensified psychiatric symptoms [11, 12].

Tobacco smoke exposure (TSE) is a significant risk factor for preventable morbidity and mortality worldwide. According to the World Health Organization (WHO), direct tobacco use or secondhand smoke exposure is responsible for more than 8 million premature deaths annually [13]. Studies have demonstrated that self-reported estimates of exposure to active and passive smoking are considerably lower than biomarker-determined exposure. Therefore, self-reported measures of TSE may not accurately reflect the true association [14].

Cotinine, the primary metabolite of nicotine, is a biomarker of tobacco exposure. Its concentration in the body is strongly correlated with tobacco consumption [15]. Cotinine is the most reliable indicator of tobacco use due to its high sensitivity, good specificity, and long half-life [16].

MR is an epidemiological method that uses genetic variants (single-nucleotide polymorphisms (SNPs)) that are associated with specific risk factors as instrumental variables (IVs) to assess possible causal associations between genetic variation and target outcomes. This method can be used to effectively reduce bias in estimates between risk factors and diseases, enhance causal inference, and overcome the limitations of observational studies [17–20].

Given the conflicting results of previous studies, further research is needed to investigate the relationship between smoke exposure and the risk of developing depression. In this study, we used an observational study perspective to analyze the association between TSE and the risk of developing depression in four dimensions: analyzed: smoking status, smoking intensity, smoking cessation duration, and serum cotinine levels. A large nationally representative sample of data was used, and various potential confounders, such as age, sex, and comorbidities, were adjusted for. Additionally, we assessed the causal effects of smoking status and cotinine level on the risk of developing depression via MR analysis.

## Materials and methods

### Overall research design

**Fig. 1:**
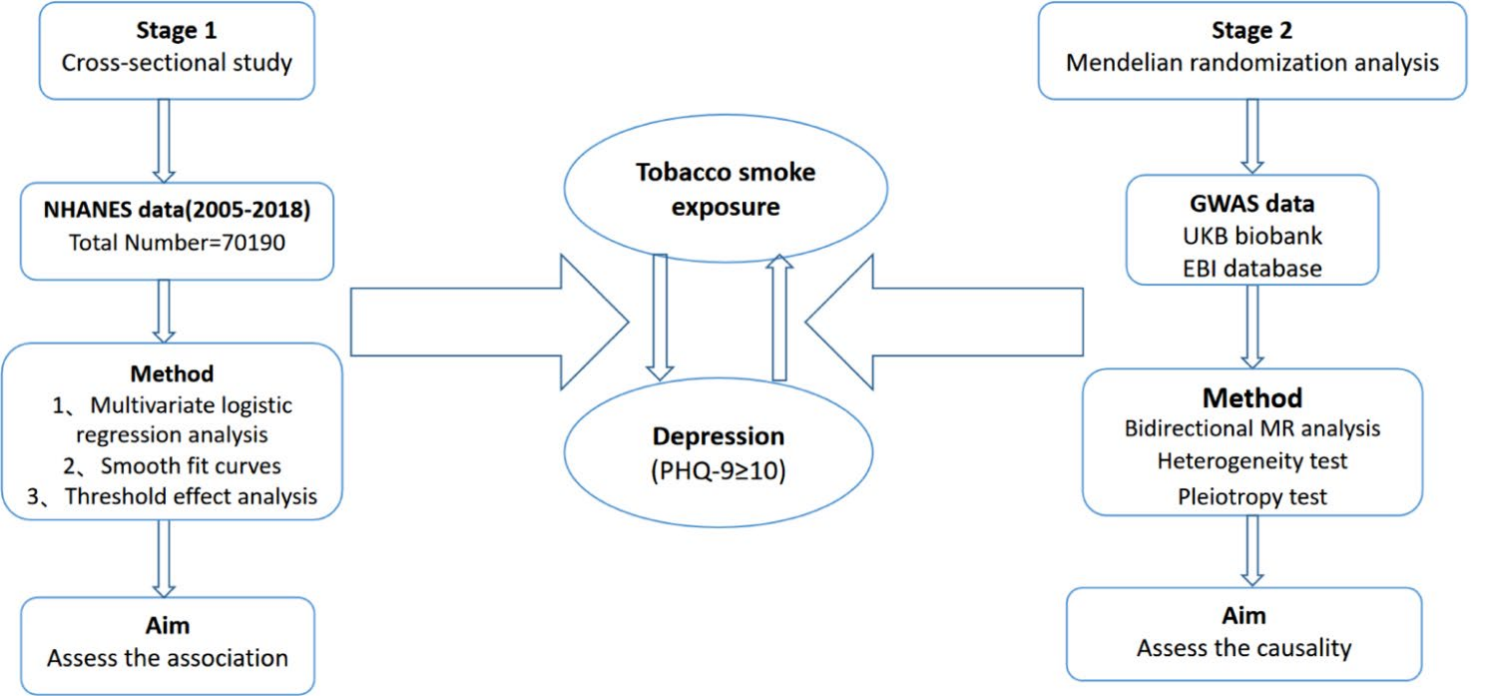
Overall study design flow chart

The research process used in this study comprised two parts (Fig. 1). First, we conducted weighted multivariate logistic regression analyses using publicly available data from the NHANES database for the years 2005–2018. The aim of this study was to identify correlations between TSE and the risk of developing depression. MR analysis utilizes summary statistics from large-scale genome-wide association studies (GWASs). GWASs are used to identify sequence variations, namely, single nucleotide polymorphisms (SNPs), across the entire human genome and to screen for SNPs associated with diseases. Using several different GWAS databases, we obtained summary statistics for smoking status (nonsmokers, former smokers, current smokers), serum cotinine levels, and depression status. We employed MR analysis to assess the causal effects of genetic determinants of smoking status and cotinine levels on the risk of developing depression.

### Observational study of NHANES

#### Data sources and study population

**Fig. 2:**
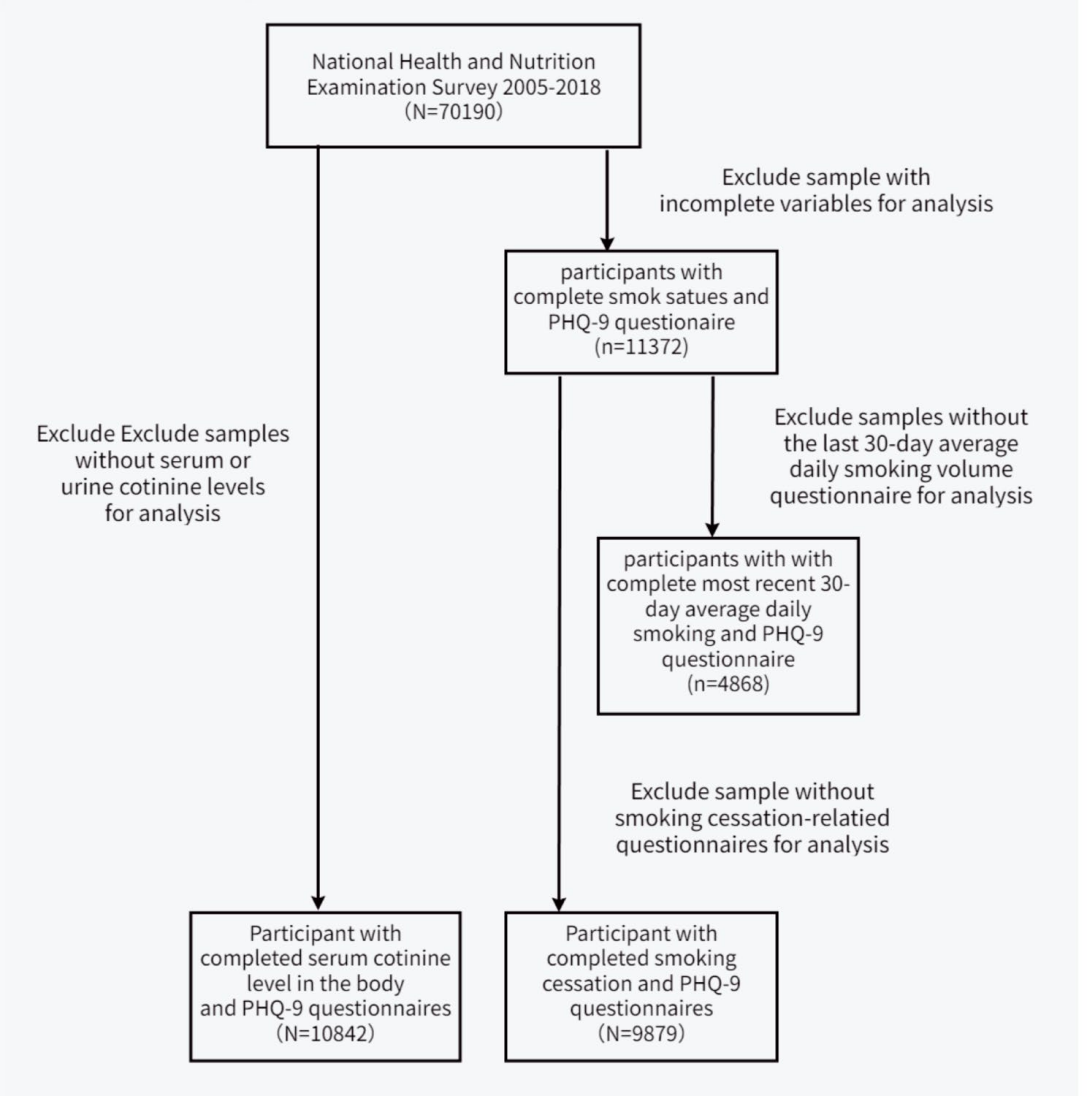
Participant screening flowchart

The NHANES is a 2-year cross-sectional health survey that evaluates the health and nutritional status of Americans in the U.S. All data in the manuscript are based on public data for secondary data analysis without ethical approval or primary data. The NHANES database started using Patient Health Questionnaire (PHQ-9) scores to evaluate depression in 2005. To analyze the impact of TSE on the risk of developing depression, we used publicly available data from participants recruited during seven NHANES waves (2005–2018), excluding participants with incomplete questionnaire information. The screening process is illustrated in Fig. 2.

#### NHANES tobacco smoke exposure assessment

We assessed TSE based on four aspects: smoking status, smoking intensity, smoking cessation duration, and serum cotinine levels. The serum cotinine concentration was determined using liquid chromatography/atmospheric pressure ionization-mass spectrometry [21]. Following similar studies [22, 23], serum cotinine levels were converted into categorical variables using 0.05 and 3.00 ng/ml as thresholds. The specific assessment criteria can be found in the supplementary materials.

#### Assessment of depression

Depression was assessed using the PHQ-9, a reliable and valid screening tool that quantifies the frequency of depressive symptoms over the past 2 weeks through 9 questions, each scored between 0 and 3 [24]. In this study, depression was defined as a PHQ-9 score of ≥ 10 [25], and nondepression was defined as a score of < 10. Previous research has demonstrated that a PHQ-9 score of ≥ 10 has a sensitivity and specificity of 88% for detecting major depression [26].

#### Assessment of covariates

We searched for factors related to smoking status, serum cotinine levels, and depression status based on previously published studies. The study included covariates such as sex (male and female), race/ethnicity (non-Hispanic white, non-Hispanic black, Mexican American, other Hispanic, or other race), age, education level (less than high school, high school, or more than high school), marital status (married/living with a partner, divorced/separated, widowed, or never married), poverty-to-income ratios (low-income:<1), alcohol consumption categories (non-drinkers; moderate drinkers: <5 alcoholic beverages per day; heavy drinkers: >5 alcoholic beverages per day), BMI (kg/m2) (classified as normal: <25; overweight: 25–30; obese: >30), and self-reported presence of diseases such as asthma, emphysema, chronic bronchitis, diabetes, hypertension, coronary heart disease, heart failure, and tumors. Participants were also asked whether they had been diagnosed with any of these conditions by a doctor.

#### Statistical analyses

The statistics were calculated taking into account the NHANES weights due to the complexity of the sampling method. The R (http://www.Rproject.org) and Empower Stats software packages (http://www.empowerstats.com) were used for all analyses. Counts and proportions were used for categorical variables, while means and standard deviations were used for continuous data. The association between smoking status and serum cotinine levelsI with the risk of developing depression were assessed using Rao-Scott chi-square tests and ttests for categorical and continuous variables, respectively. We used weighted multivariate logistic regression to evaluate the associations of smoking status and serum cotinine level with the risk of developing depression. The extent of the association was determined using the odds ratio (OR) and 95% confidence intervals (95% CI), respectively, with *p* < 0.05 considered to indicate statistical significance.

### Mendelian randomization study

#### Data sources

The MR analyses used data obtained from publicly available summary statistics of large-scale GWAS datasets. GWAS data on smoking status (never smokers, former smokers, and current smokers) were obtained from the UK Biobank database (https://www.ukbiobank.ac.uk). Cotinine levels GWAS data were obtained from the EBI database (https://www.ebi.ac.uk), and genetic information on the risk of developing depression was obtained from the IEU Open GWAS database (https://gwas.mrcieu.ac.uk) [27]. Table S1 provides specific information. All analyses conducted in this study are based on public data that are available for secondary data analysis without ethical approval or primary data.

#### MR analysis

**Fig. 3:**
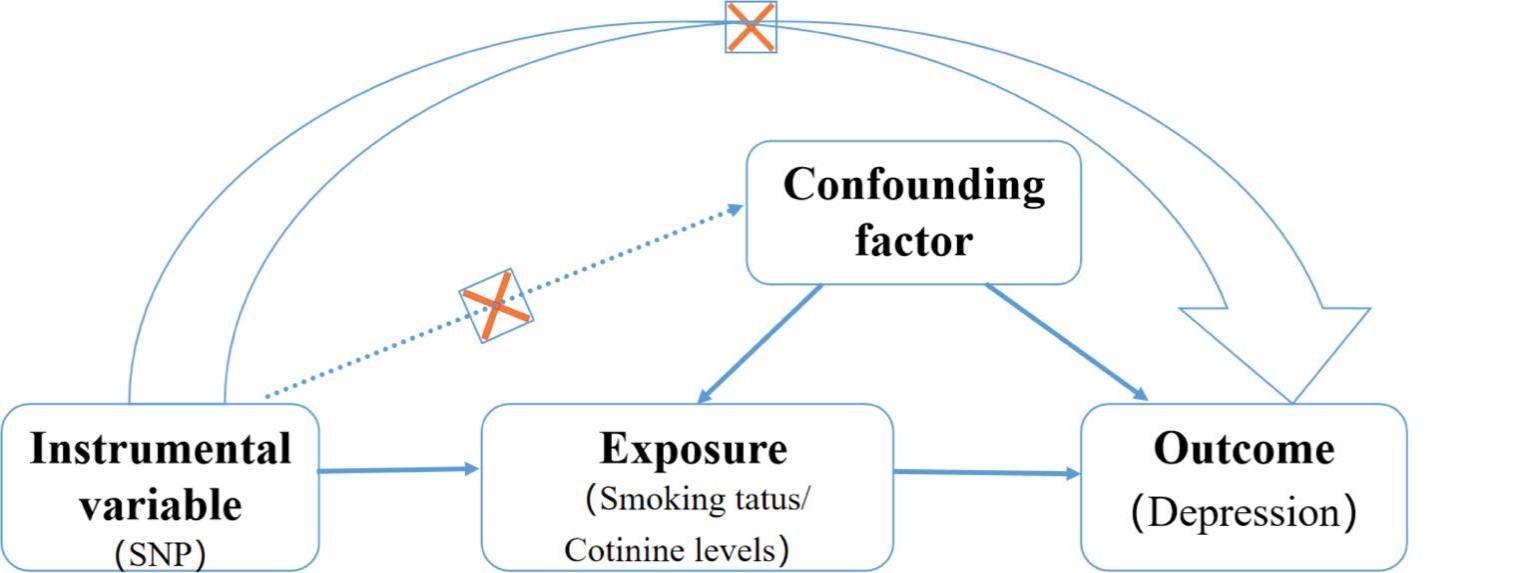
MR analysis flow chart

To conduct MR analyses, IVs must satisfy three assumptions: (1) correlation assumption, which means that genetic variants used as IVs are associated with exposure; (2) exclusivity assumption, which means that genetic variants used as IVs affect outcomes only through exposure; (3) independence assumption, which means that genetic variants are not associated with confounders related to any potential influences on exposure and outcomes [28](Fig. 3).

Moreover, in this study, IVs were required to meet four specific selection criteria. (1) The thresholds for genetic variant SNP loci for smoking status and cotinine level were set at *P* < 5 × 10^− 8^(smoking status)/*P* < 5 × 10^− 6^(cotinine level). (2) The independence of SNP loci was assessed based on linkage disequilibrium. IVs without linkage effects were screened from the smoking status and cotinine level data (the parameters were set at r2 < 0.001, kb = 10,000) [29]. Then, instrumental variables without linkage effects were excluded from the data of instrumental variables that were correlated with the risk of developing depression. (3) The data for exposure and outcome were combined and processed to remove palindromes and unmatched SNPs. (4) We computed the F-statistic to validate the strength of individual SNPs and mitigate the effect of potential bias. To avoid the effect of weak instrumental bias on causal inference, we used the Formula F = β^2^/se^2^ to calculate the strength of instrumental variables (IVs) and eliminated IVs with F < 10 [30].

#### Statistical analysis

The analyses primarily relied on the inverse-variance weighting (IVW) method to evaluate the causal effects of smoking status and cotinine levels on the risk of developing depression. Additionally, MR‒Egger, the weighted median, the weighted mode, and the simple mode were used to validate the reliability of the IVW results [31].

IVW and MR‒Egger were used to perform heterogeneity tests in MR sensitivity analyses to test for differences between individual IVs. Heterogeneity was quantified by the p-value of Cochran’s Q test. If *p* < 0.05, the results were not heterogeneous. However, the presence of heterogeneity did not affect the reliability of the IVW results. Potential horizontal pleiotropy was assessed using MR-Egger and MR‒PRESSO based on intercept terms. The results showed no evidence of horizontal pleiotropy (*P* > 0.05) [32]. Leave-one-out analyses were also conducted to check for any single SNP that significantly drove the IVW estimates. Additionally, the reliability of the results was assessed by looking for asymmetry in the funnel plots. All analyses were performed using the TwoSampleMR and MR‒PRESSO packages in R software (version 4.2.1) [33].

## Results

### NHANES observational study

#### Smoking status

##### Basic characteristics of participants’ smoking behavior

A total of 116,876 US residents participated in seven rounds of the NHANES survey between 2005 and 2018. After the data were screened, 11,372 participants were included to assess the interrelationship between smoking status and the risk of developing depression. The mean age of the participants was 45.89 ± 17.09 years, with 6,109 (53.71%) males and 5,263 (46.29%) females.

The study participants were categorized into three groups based on their smoking status. Of the total participants, 49.59% (*n* = 5640) were nonsmokers, 25.04% (*n* = 2848) were ex-smokers, and 25.37% (*n* = 2884) were current smokers. Table 1 displays their clinical characteristics. Current smokers were younger and predominantly male, non-Hispanic white, and non-Hispanic black. Additionally, they had lower BMIs, PIRs, and education levels than did the other two groups. The study revealed that participants had a greater likelihood of consuming alcohol, residing with their parents, and experiencing health conditions such as asthma, emphysema, chronic bronchitis, and depression. The study participants were divided into two groups based on their PHQ-9 scores. Of the total participants, 54.8% (*n* = 10,451) were classified as nondepressed (PHQ-9 < 10) and 54.8% (*n* = 921) were classified as depressed (PHQ-9 ≥ 10). Table S2 shows the clinical characteristics of both groups.

**Table 1:** Baseline characteristics of participants by smoking status

Characteristics	Smoking status				*P*-value
	Total (*N* = 11,372)	Never smokers (*N* = 5639)	Previous smokers (*N* = 2848)	Current smokers (*N* = 2885)	
Age(year), Median(95%CI)	45.89 ± 17.09	43.56 ± 16.62	54.62 ± 17.21	41.83 ± 14.70	< 0.001
Gender, *n* (%)					< 0.001
Male	6108 (53.71%)	2659 (47.14%)	1730 (60.74%)	1720 (59.63%)	
Female	5264 (46.28%)	2981 (52.85%)	1118 (39.25%)	1164 (40.36%)	
Race/ethnicity, *n* (%)					< 0.001
Mexican American	1730 (15.21%)	975 (17.28%)	398 (13.97%)	357 (12.37%)	
Other Hispanic	932 (8.19%)	529 (9.37%)	195 (6.84%)	208 (7.21%)	
Non-Hispanic White	5740 (50.47%)	2545 (45.12%)	1740 (61.09%)	1455 (50.45%)	
Non-Hispanic Black	2269 (19.95%)	1178 (20.88%)	379 (13.30%)	712 (24.68%)	
Other race	701 (6.16%)	413 (7.32%)	136 (4.77%)	152 (5.27%)	
Education, *n* (%)					< 0.001
Below high school	2415 (21.23%)	966 (17.12%)	546 (19.17%)	903 (31.31%)	
High school	2556 (22.47%)	1084 (19.21%)	609 (21.38%)	863 (29.92%)	
Above high school	6401 (56.28%)	3590 (63.65%)	1693 (59.44%)	1118 (38.76%)	
Marital, *n* (%)					< 0.001
Married/Partner	6932 (60.95%)	3477 (61.64%)	1956 (68.67%)	1499 (51.97%)	
Widowed	569 (5.00%)	248 (4.39%)	207 (7.26%)	114 (3.95%)	
Divorced/Separated	1605 (14.11%)	643 (11.40%)	396 (13.90%)	566 (19.62%)	
Never married	2266 (19.92%)	1272 (22.55%)	289 (10.14%)	705 (24.44%)	
PIR, n (%)					< 0.001
<1.3	3050(26.82%)	1256 (22.26%)	562 (19.73%)	1232 (42.71%)	
1.3–3.5	4121(36.23%)	2030 (35.99%)	1062 (37.28%)	1029 (35.67%)	
> 3.5	4201(36.94%)	2354 (41.73%)	1224 (42.97%)	623 (21.60%)	
BMI, n (%)					< 0.001
< 25	2753(24.20%)	1315 (23.31%)	527 (18.50%)	911 (31.58%)	
25–30	4735(41.63%)	2344 (41.56%)	1275 (44.76%)	1116 (38.69%)	
>30	3884(34.15%)	1981 (35.12%)	1046 (36.72%)	857 (29.71%)	
Drinking status, n (%)					< 0.001
No drink	3893 (34.23%)	2322 (41.17%)	1080 (37.92%)	491 (17.02%)	
Moderate alcohol consumption	6040 (53.11%)	2811 (49.84%)	1510 (53.01%)	1719 (59.60%)	
Excessive alcohol consumption	1439 (12.65%)	507 (8.98%)	258 (9.05%)	674 (23.37%)	
Diabetes, n(%)					< 0.001
YES	940 (8.26%)	410 (7.26%)	335 (11.76%)	195 (6.76%)	
NO	10,432 (91.73%)	5230 (92.73%)	2513 (88.23%)	2689 (93.23%)	
Hypertension, *n* (%)					< 0.001
YES	3374 (29.66%)	1467 (26.01%)	1150 (40.37%)	757 (26.24%)	
NO	7998 (70.33%)	4173 (73.98%)	1698 (59.62%)	2127 (73.75%)	
Asthma, *n* (%)					< 0.001
YES	1609 (14.14%)	756 (13.40%)	370 (12.99%)	483 (16.74%)	
NO	9763 (85.85%)	4884 (86.59%)	2478 (87.00%)	2401 (83.25%)	
Heart failure, n (%)					< 0.001
YES	215 (1.89%)	67 (1.18%)	94 (3.30%)	54 (1.87%)	
NO	11,157 (98.10%)	5573 (98.81%)	2754 (96.69%)	2830 (98.12%)	
Coronary heart disease, n (%)					< 0.001
YES	353 (3.10%)	108 (1.91%)	182 (6.39%)	63 (2.18%)	
NO	11,019 (96.89%)	5532 (98.08%)	2666 (93.60%)	2821 (97.81%)	
Emphysema, n (%)					< 0.001
YES	193 (1.69%)	8 (0.14%)	86 (3.01%)	99 (3.43%)	
NO	11,179 (98.30%)	5632 (99.85%)	2762 (96.98%)	2785 (96.56%)	
Chronic bronchitis, n (%)					< 0.001
YES	588 (5.17%)	178 (3.15%)	170 (5.96%)	240 (8.32%)	
NO	10,784 (94.82%)	5462 (96.84%)	2678 (94.03%)	2644 (91.67%)	
Cancer, n (%)					< 0.001
YES	940 (8.26%)	372 (6.59%)	401 (14.08%)	167 (5.79%)	
NO	10,432 (91.73%)	5268 (93.40%)	2447 (85.91%)	2717 (94.20%)	
Depression, n(%)					< 0.001
YES	10,451(91.90%)	5316 (94.25%)	2665 (93.57%)	2470 (85.64%)	
NO	921(8.09%)	324 (5.74%)	183 (6.42%)	414 (14.35%)	

##### Association between smoking status and depression

This study utilized multivariable logistic regression analysis to construct three models to examine the association between smoking status and depression (Table 2). The research findings indicate that, both in the unadjusted and adjusted models, compared to nonsmokers and former smokers, current smokers have a 1.94-fold increased risk of developing depression, showing a positive correlation with the risk of developing depression (OR 1.94; 95% CI 1.64–2.31; *P* < 0.001).

**Table 2:** Weighted multivariate adjusted logistic regression of smoking status and depression risk

Exposure	Unadjusted model	Adjust moedel I	Adjust model II
Smoking status, category			
Never smokers	1.00	1.00	1.00
Previous smokers	1.13 (0.93, 1.36)	1.34 (1.10, 1.63)	1.18 (0.96, 1.44)
Current smokers	2.75 (2.36, 3.20) *P*<0.001	2.18 (1.85, 2.58) *P*<0.001	1.94 (1.64, 2.31) *P*<0.001
Smoking status continuous	1.67 (1.54, 1.81) *P*<0.001	1.48 (1.36, 1.61) *P*<0.001	1.39 (1.28, 1.52) *P*<0.001
P for trend	*P*<0.001	*P*<0.001	*P*<0.001

#### Smoking intensity

##### Basic characteristics of participants’ smoking intensity

We identified 4868 individuals who provided complete daily smoking data for the past 30 days through screening of participant smoking intensity-related data. The participants were then categorized into four groups (Q1-Q4) based on their daily smoking data. The clinical characteristics of each group are shown in Table 3. The Q4 group, which had the highest number of daily cigarettes, was found to be older, with fewer females, a greater preference for alcohol, and a greater proportion of non-Hispanic whites than the other three groups. The study revealed that individuals with lower levels of education were more likely to have asthma, diabetes, hypertension, tumors, emphysema, chronic bronchitis, and coronary heart disease. The clinical characteristics of smoking intensity for participants with non-depression or depression are presented in Table S3.

**Table 3:** Baseline characteristics of participants by Smoking volume

Characteristics	Smoking volume					*P*-value
	Total (*N* = 4868)	Q1 (*N* = 975)	Q2 (*N* = 1121)	Q3 (*N* = 1444)	Q4 (*N* = 1328)	
Age(year), Median(95%CI)	42.6565 ± 14.71	39.7026 ± 14.62	41.5129 ± 15.11	43.1247 ± 14.79	45.2816 ± 13.85	< 0.001
Gender, *n* (%)						< 0.001
Male	2871 (58.97%)	606 (62.15%)	618 (55.12%)	809 (56.02%)	838 (63.10%)	
Female	1997 (41.02%)	369 (37.84%)	503 (44.87%)	635 (43.97%)	490 (36.89%)	
Race/ethnicity, *n* (%)						< 0.001
Mexican American	541 (11.11%)	231 (23.69%)	155 (13.82%)	105 (7.27%)	50 (3.76%)	
Other Hispanic	336 (6.90%)	109 (11.17%)	91 (8.11%)	83 (5.74%)	53 (3.99%)	
Non-Hispanic White	2368 (48.64%)	289 (29.64%)	361 (32.20%)	759 (52.56%)	959 (72.21%)	
Non-Hispanic Black	1247 (25.61%)	256 (26.25%)	426 (38.00%)	379 (26.24%)	186 (14.00%)	
Other race	376 (7.72%)	90 (9.23%)	88 (7.85%)	118 (8.17%)	80 (6.02%)	
Education, *n* (%)						< 0.001
Below high school	1366 (28.06%)	293 (30.05%)	299 (26.67%)	372 (25.76%)	402 (30.27%)	
High school	1476 (30.32%)	233 (23.89%)	328 (29.25%)	461 (31.92%)	454 (34.18%)	
Above high school	2026 (41.61%)	449 (46.05%)	494 (44.06%)	611 (42.31%)	472 (35.54%)	
Marital, *n* (%)						< 0.001
Married/Partner	2481 (50.96%)	506 (51.89%)	547 (48.79%)	722 (50.00%)	706 (53.16%)	
Widowed	206 (4.23%)	33 (3.38%)	45 (4.01%)	66 (4.57%)	62 (4.66%)	
Divorced/Separated	971 (19.94%)	160 (16.41%)	196 (17.48%)	305 (21.12%)	310 (23.34%)	
Never married	1210 (24.85%)	276 (28.30%)	333 (29.70%)	351 (24.30%)	250 (18.82%)	
PIR, n (%)						0.035
<1.3	2142 (44.00%)	402 (41.23%)	490 (43.71%)	618 (42.79%)	632 (47.59%)	
1.3–3.5	1807 (37.12%)	374 (38.35%)	434 (38.71%)	548 (37.95%)	451 (33.96%)	
> 3.5	919 (18.87%)	199 (20.41%)	197 (17.57%)	278 (19.25%)	245 (18.44%)	
BMI, n (%)						0.033
< 25	1489 (30.58%)	263 (26.97%)	335 (29.88%)	456 (31.57%)	435 (32.75%)	
25–30	1812 (37.22%)	365 (37.43%)	411 (36.66%)	542 (37.53%)	494 (37.19%)	
>30	1567 (32.18%)	347 (35.58%)	375 (33.45%)	446 (30.88%)	399 (30.04%)	
Drinking status, n (%)						< 0.001
No drink	860 (17.66%)	166 (17.02%)	196 (17.48%)	270 (18.69%)	228 (17.16%)	
Moderate alcohol consumption	2955 (60.70%)	615 (63.07%)	710 (63.33%)	884 (61.21%)	746 (56.17%)	
Excessive alcohol consumption	1053 (21.63%)	194 (19.89%)	215 (19.17%)	290 (20.08%)	354 (26.65%)	
Diabetes, n(%)						0.03
YES	377 (7.74%)	64 (6.56%)	80 (7.13%)	106 (7.34%)	127 (9.56%)	
NO	4491 (92.25%)	911 (93.43%)	1041 (92.86%)	1338 (92.65%)	1201 (90.43%)	
Hypertension, *n* (%)						< 0.001
YES	1429 (29.35%)	235 (24.10%)	329 (29.34%)	426 (29.50%)	439 (33.05%)	
NO	3439 (70.64%)	740 (75.89%)	792 (70.65%)	1018 (70.49%)	889 (66.94%)	
Asthma, *n* (%)						0.002
YES	849 (17.44%)	134 (13.74%)	185 (16.50%)	276 (19.11%)	254 (19.12%)	
NO	4019 (82.55%)	841 (86.25%)	936 (83.49%)	1168 (80.88%)	1074 (80.87%)	
Heart failure, n (%)						0.017
YES	99 (2.03%)	8 (0.82%)	30 (2.67%)	30 (2.07%)	31 (2.33%)	
NO	4769 (97.96%)	967 (99.17%)	1091 (97.32%)	1414 (97.92%)	1297 (97.66%)	
Coronary heart disease, n (%)						0.004
YES	114 (2.34%)	13 (1.33%)	20 (1.78%)	35 (2.42%)	46 (3.46%)	
NO	4754 (97.65%)	962 (98.66%)	1101 (98.21%)	1409 (97.57%)	1282 (96.53%)	
Emphysema, n (%)						< 0.001
YES	160 (3.28%)	12 (1.23%)	19 (1.69%)	45 (3.11%)	84 (6.32%)	
NO	4708 (96.71%)	963 (98.76%)	1102 (98.30%)	1399 (96.88%)	1244 (93.67%)	
Chronic bronchitis, n (%)						< 0.001
YES	435 (8.93%)	44 (4.51%)	78 (6.95%)	145 (10.04%)	168 (12.65%)	
NO	4433 (91.06%)	931 (95.48%)	1043 (93.04%)	1299 (89.95%)	1160 (87.34%)	
Cancer, n (%)						0.007
YES	312 (6.40%)	47 (4.82%)	61 (5.44%)	97 (6.71%)	107 (8.05%)	
NO	4556 (93.59%)	928 (95.17%)	1060 (94.55%)	1347 (93.28%)	1221 (91.94%)	
Depression, n(%)						< 0.001
YES	4147 (85.18%)	862 (88.41%)	983 (87.68%)	1227 (84.97%)	1075 (80.94%)	
NO	721 (14.81%)	113 (11.58%)	138 (12.31%)	217 (15.02%)	253 (19.05%)	

##### Association between smoking intensity and depression

Multivariate logistic regression analysis revealed that the association between smoking intensity and the risk of developing depression persisted (Table 4). The study revealed that the risk of developing depression increased as smoking frequency increased. Smokers in the Q4 group, who smoked the most, had a 1.66-fold greater risk of developing depression than did those in the Q1 group (OR 1.66; 95% CI 1.26–2.17; *P* < 0.01).

**Table 4:** Weighted multivariate adjusted logistic regression of smoking volume and depression risk

Exposure	Unadjusted model	Adjust moedel I	Adjust model II
Smoking volume, category			
Q1	1	1	1
Q2	1.07 (0.82, 1.39) 0.61	1.01 (0.76, 1.33) 0.93	0.96 (0.72, 1.27) 0.78
Q3	1.34 (1.05, 1.72) 0.01	1.32 (1.02, 1.71) 0.03	1.23 (0.95, 1.61) 0.11
Q4	1.79 (1.4134, 2.2804) 0.01	1.8671 (1.4334, 2.4319) 0.01	1.65 (1.26, 2.17) 0.01
Smoking volume Continuous	1.02 (1.01, 1.03) < 0.001	1.02 (1.01, 1.03) < 0.001	1.02 (1.01, 1.03) < 0.001
P for trend	*P*<0.001	*P*<0.001	*P*<0.001

#### Smoking cessation duration

##### Basic characteristics of participants’ smoking cessation duration

In this study, we analyzed a total of 9879 participants, who were categorized into four groups (Q1-Q4) based on the duration of smoking cessation. The Q4 group, which had the longest smoking cessation duration, was found to be older, predominantly male, mostly non-Hispanic white, and had higher education, stable marital status, and higher income than the other three groups. Additionally, Table 5 shows a higher prevalence of diabetes, hypertension, tumors, heart failure and depression in this group. Additionally, the clinical characteristics of smoking cessation duration for individuals with and without depression are shown in Table S4. tumors3.1.3.2 Association between smoking cessation duration and depression.

**Table 5:** Baseline characteristics of participants by smoking cessation duration

Characteristics	Smoking cessation duration					*P*-value
	Total (*N* = 9879)	Q1 (*N* = 5871)	Q2 (*N* = 1698)	Q3 (*N* = 1129)	Q4 (*N* = 1181)	
Age(year), Median(95%CI)	48.469 ± 16.89	42.342 ± 14.85	48.80 ± 15.68	59.19 ± 12.66	68.19 ± 9.85	< 0.001
Gender, *n* (%)						< 0.001
Male	5922 (59.94%)	3472 (59.13%)	994 (58.53%)	675 (59.78%)	781 (66.13%)	
Female	3957 (40.05 %)	2399 (40.86%)	704 (41.46%)	454 (40.21%)	400 (33.86%)	
Race/ethnicity, *n* (%)						< 0.001
Mexican American	1248 (12.63%)	735 (12.51%)	255 (15.01%)	147 (13.02%)	111 (9.39%)	
Other Hispanic	772 (7.81%)	428 (7.29%)	143 (8.42%)	114 (10.09%)	87 (7.36%)	
Non-Hispanic White	5142 (52.04%)	2836 (48.30%)	909 (53.53%)	623 (55.18%)	774 (65.53%)	
Non-Hispanic Black	1944 (19.67%)	1393 (23.72%)	239 (14.07%)	168 (14.88%)	144 (12.19%)	
Other race	773 (7.82%)	479 (8.15%)	152 (8.95%)	77 (6.82%)	65 (5.50%)	
Education, *n* (%)						< 0.001
Below high school	2270 (22.97%)	1567 (26.69%)	322 (18.96%)	198 (17.53%)	183 (15.49%)	
High school	2590 (26.21%)	1716 (29.22%)	367 (21.61%)	270 (23.91%)	237 (20.06%)	
Above high school	5019 (50.80%)	2588 (44.08%)	1009 (59.42%)	661 (58.54%)	761 (64.43%)	
Marital, *n* (%)						< 0.001
Married/Partner	5847 (59.18%)	3067 (52.23%)	1158 (68.19%)	784 (69.44%)	838 (70.95%)	
Widowed	547 (5.53%)	237 (4.03%)	78 (4.59%)	87 (7.70%)	145 (12.27%)	
Divorced/Separated	1721 (17.42%)	1132 (19.28%)	258 (15.19%)	180 (15.94%)	151 (12.78%)	
Never married	1764 (17.85%)	1435 (24.44%)	204 (12.01%)	78 (6.90%)	47 (3.97%)	
PIR, n (%)						< 0.001
<1.3	3229 (32.68%)	2468 (42.03%)	398 (23.43%)	196 (17.36%)	167 (14.14%)	
1.3–3.5	3706 (37.51%)	2168 (36.92%)	657 (38.69%)	424 (37.55%)	457 (38.69%)	
> 3.5	2944 (29.80%)	1235 (21.03%)	643 (37.86%)	509 (45.08%)	557 (47.16%)	
BMI, n (%)						< 0.001
< 25	2381 (24.10%)	1704 (29.02%)	320 (18.84%)	169 (14.96%)	188 (15.91%)	
25–30	3963 (40.11%)	2224 (37.88%)	677 (39.87%)	493 (43.66%)	569 (48.17%)	
>30	3535 (35.78%)	1943 (33.09%)	701 (41.28%)	467 (41.36%)	424 (35.90%)	
Drinking status, n (%)						< 0.001
No drink	2685 (27.17%)	1097 (18.68%)	509 (29.97%)	476 (42.16%)	603 (51.05%)	
Moderate alcohol consumption	5696 (57.65%)	3564 (60.70%)	1001 (58.95%)	585 (51.81%)	546 (46.23%)	
Excessive alcohol consumption	1498 (15.16%)	1210 (20.60%)	188 (11.07%)	68 (6.02%)	32 (2.70%)	
Diabetes, n(%)						< 0.001
YES	1074 (10.87%)	469 (7.98%)	224 (13.19%)	186 (16.47%)	195 (16.51%)	
NO	8805 (89.12%)	5402 (92.01%)	1474 (86.80%)	943 (83.52%)	986 (83.48%)	
Hypertension, *n* (%)						< 0.001
YES	3487 (35.29%)	1698 (28.92%)	609 (35.86%)	534 (47.29%)	646 (54.69%)	
NO	6392 (64.70%)	4173 (71.07%)	1089 (64.13%)	595 (52.70%)	535 (45.30%)	
Asthma, *n* (%)						< 0.001
YES	1555 (15.74%)	999 (17.01%)	260 (15.31%)	146 (12.93%)	150 (12.70%)	
NO	8324 (84.25%)	4872 (82.98%)	1438 (84.68%)	983 (87.06%)	1031 (87.29%)	
Heart failure, n (%)						< 0.001
YES	286 (2.89%)	130 (2.21%)	62 (3.65%)	39 (3.45%)	55 (4.65%)	
NO	9593 (97.10%)	5741 (97.78%)	1636 (96.34%)	1090 (96.54%)	1126 (95.34%)	
Coronary heart disease, n (%)						< 0.001
YES	424 (4.29%)	154 (2.62%)	77 (4.53%)	80 (7.08%)	113 (9.56%)	
NO	9455 (95.70%)	5717 (97.37%)	1621 (95.46%)	1049 (92.91%)	1068 (90.43%)	
Emphysema, n (%)						0.937
YES	300 (3.03%)	183 (3.11%)	50 (2.94%)	34 (3.01%)	33 (2.79%)	
NO	9579 (96.96%)	5688 (96.88%)	1648 (97.05%)	1095 (96.98%)	1148 (97.20%)	
Chronic bronchitis, n (%)						< 0.001
YES	745 (7.54%)	497 (8.46%)	98 (5.77%)	76 (6.73%)	74 (6.26%)	
NO	9134 (92.45%)	5374 (91.53%)	1600 (94.22%)	1053 (93.26%)	1107 (93.73%)	
Cancer, n (%)						< 0.001
YES	1010 (10.22%)	365 (6.21%)	161 (9.48%)	183 (16.20%)	301 (25.48%)	
NO	8869 (89.77%)	5506 (93.78%)	1537 (90.51%)	946 (83.79%)	880 (74.51%)	
Depression, n(%)						< 0.001
YES	8798 (89.05%)	5046 (85.94%)	1564 (92.10%)	1062 (94.06%)	1126 (95.34%)	
NO	1081 (10.94%)	825 (14.05%)	134 (7.89%)	67 (5.93%)	55 (4.65%)	

The relationship between smoking cessation duration and the risk of developing depression was analyzed by adjusting for various confounding factors (Table 6). All models demonstrated that quitting smoking was linked to a decreased risk of developing depression (all models, P for trend < 0.001). In Model II, compared to the Q1 group with the shortest smoking cessation duration, the Q4 group with the longest smoking cessation duration showed a 52% reduction in the risk of developing depression.

**Table 6:** Weighted multivariate adjusted logistic regression of smoking cessation duration and depression risk

Exposure	Unadjusted model	Adjust moedel I	Adjust model II
Smoking cessation duration category			
Q1	1	1	1
Q2	0.52 (0.43, 0.63) < 0.001	0.65 (0.53, 0.80) < 0.001	0.65 (0.53, 0.80) < 0.001
Q3	0.38 (0.29, 0.49) < 0.001	0.52 (0.39, 0.68) < 0.001	0.52 (0.39, 0.69) < 0.001
Q4	0.29 (0.22, 0.39) < 0.001	0.44 (0.32, 0.60) < 0.001	0.48 (0.34, 0.66) < 0.001
Smoking cessation duration Continuous	0.96 (0.95, 0.96) < 0.001	0.97 (0.96, 0.98) < 0.001	0.97 (0.96, 0.98) < 0.001
P for trend	*P*<0.001	*P*<0.001	*P*<0.001

##### Smoothed fitted curves and threshold effect analysis

**Fig. 4:**
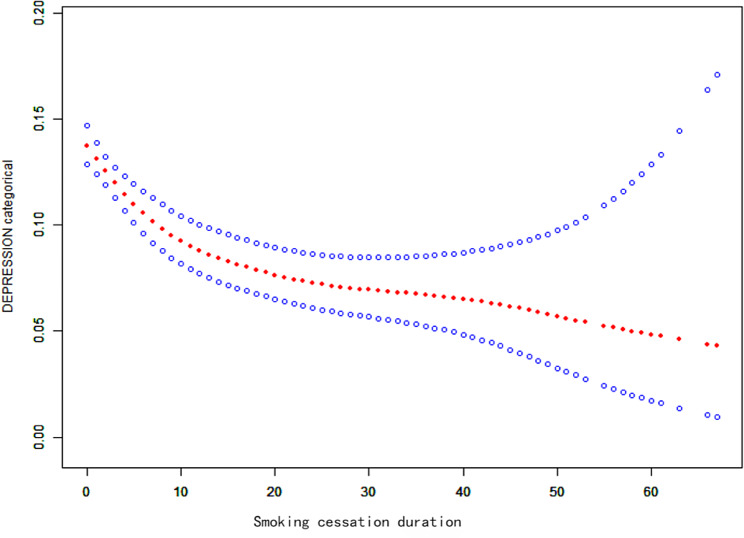
Smooth curve fit graph (solid red line represents smooth curve fit between variables, blue band represents 95% confidence interval of fit)

We found a nonlinear relationship between smoking cessation duration and the risk of developing depression through smooth fitted curves. The results indicate that as smoking cessation duration increases, the risk of developing depression continuously decreases (Fig. 4). The two-stage linear regression model results indicate that the inflection point for the threshold effect between the time to quit smoking and the risk of developing depression was 4 years (Table 7). This study revealed that smoking cessation duration can reduce the risk of developing depression. Compared to those with a smoking cessation duration of less than 4 years or more than 4 years, the risk of depression onset decreased with each additional year of smoking cessation, but overall, the risk of depression onset steadily decreased with increasing smoking cessation duration (*P* < 0.01).

**Table 7:** Threshold effect analysis

Exposure	Adjusted OR (95% CI), *P*-value
Smoking cessation duration	
Inflection point(K)	4
Smoking cessation duration < 21.5	0.87 (0.82, 0.91) < 0.001
Smoking cessation duration ≥ 21.5	0.98 (0.97, 0.99) 0.01
Log likelihood ratio	0.001

#### Serum cotinine levels

##### Basic characteristics of participants’ serum cotinine levels

A total of 10,842 participants participated in this study. The relationship between tobacco smoke exposure and the risk of developing depression, including serum cotinine levels, was analyzed from various perspectives. Table 8 displays the clinical characteristics of the participants based on their serum cotinine levels. The group with the highest cotinine levels (T3) consisted mostly of non-Hispanic white and black males with lower levels of education compared to the other two groups. They were predominantly unmarried or divorced/separated, had low household incomes, and had a higher prevalence of asthma, emphysema, chronic bronchitis, and depression. They were predominantly unmarried or divorced/separated, had low household incomes, and had a higher prevalence of asthma, emphysema, chronic bronchitis, and depression. Alcohol consumption was also more common in this group. They were predominantly unmarried or divorced/separated, had low household incomes, and had a higher prevalence of asthma, emphysema, chronic bronchitis, and depression. The clinical characteristics of patients with different serum cotinine levels in the depression group and nondepression group are presented in Table S5.

**Table 8:** Baseline characteristics of participants by Serum cotinine levels

Characteristics	Serum cotinine levels				*P*-value
	Total (*N* = 10,843)	T1 (*N* = 4588)	T2 (*N* = 2846)	T3 (*N* = 3409)	
Age(year), Median(95%CI)	45.99 ± 17.08	49.96 ± 17.33	44.53 ± 17.45	41.87 ± 15.15	< 0.001
Gender, *n* (%)					< 0.001
Male	5836 (53.82%)	2140 (46.64%)	1577 (55.41%)	2119 (62.17%)	< 0.001
Female	5006 (46.17%)	2448 (53.35%)	1269 (44.58%)	1289 (37.82%)	
Race/ethnicity, *n* (%)					
Mexican American	1679 (15.48%)	839 (18.28%)	478 (16.79%)	362 (10.62%)	< 0.001
Other Hispanic	893 (8.23%)	442 (9.63%)	223 (7.83%)	228 (6.69%)	
Non-Hispanic White	5529 (50.99%)	2446 (53.31%)	1326 (46.59%)	1757 (51.55%)	
Non-Hispanic Black	2082 (19.20%)	544 (11.85%)	646 (22.69%)	892 (26.17%)	
Other race	659 (6.07%)	317 (6.90%)	173 (6.07%)	169 (4.95%)	
Education, *n* (%)					
Below high school	2298 (21.19%)	673 (14.66%)	625 (21.96%)	1000 (29.34%)	< 0.001
High school	2430 (22.41%)	786 (17.13%)	635 (22.31%)	1009 (29.60%)	
Above high school	6114 (56.39%)	3129 (68.19%)	1586 (55.72%)	1399 (41.05%)	
Marital, *n* (%)					
Married/Partner	6658 (61.40%)	3219 (70.16%)	1650 (57.97%)	1789 (52.49%)	< 0.001
Widowed	540 (4.98%)	258 (5.62%)	153 (5.37%)	129 (3.78%)	
Divorced/Separated	1522 (14.03%)	486 (10.59%)	391 (13.73%)	645 (18.92%)	
Never married	2122 (19.57%)	625 (13.62%)	652 (22.90%)	845 (24.79%)	
PIR, n (%)					
<1.3	2887 (26.62%)	725 (15.80%)	782 (27.47%)	1380 (40.49%)	< 0.001
1.3–3.5	3932 (36.26%)	1623 (35.37%)	1076 (37.80%)	1233 (36.17%)	
> 3.5	4023 (37.10%)	2240 (48.82%)	988 (34.71%)	795 (23.32%)	
BMI, n (%)					
< 25	2605 (24.02%)	1068 (23.27%)	529 (18.58%)	1008 (29.56%)	< 0.001
25–30	4529 (41.76%)	2033 (44.31%)	1161 (40.79%)	1335 (39.16%)	
>30	3709 (34.20%)	1487 (32.41%)	1156 (40.61%)	1066 (31.27%)	
Drinking status, n (%)					
No drink	3714 (34.25%)	2195 (47.84%)	903 (31.72%)	616 (18.07%)	0.014
Moderate alcohol consumption	5769 (53.20%)	2136 (46.55%)	1570 (55.16%)	2063 (60.53%)	
Excessive alcohol consumption	1359 (12.53%)	257 (5.60%)	373 (13.10%)	729 (21.39%)	
Diabetes, n(%)					< 0.001
YES	889 (8.19%)	396 (8.63%)	252 (8.85%)	241 (7.06%)	
NO	9954 (91.80%)	4192 (91.36%)	2594 (91.14%)	3168 (92.93%)	
Hypertension, *n* (%)					< 0.001
YES	3208 (29.58%)	1442 (31.42%)	870 (30.56%)	896 (26.29%)	
NO	7634 (70.41%)	3146 (68.57%)	1976 (69.43%)	2512 (73.70%)	
Asthma, *n* (%)					0.062
YES	1521 (14.02%)	554 (12.07%)	407 (14.30%)	560 (16.42%)	
NO	9322 (85.97%)	4034 (87.92%)	2439 (85.69%)	2849 (83.57%)	
Heart failure, n (%)					0.054
YES	207 (1.90%)	71 (1.54%)	62 (2.17%)	74 (2.17%)	
NO	10,635 (98.09%)	4517 (98.45%)	2784 (97.82%)	3334 (97.82%)	
Coronary heart disease, n (%)					< 0.001
YES	338 (3.11%)	162 (3.53%)	88 (3.09%)	88 (2.58%)	
NO	10,504 (96.88%)	4426 (96.46%)	2758 (96.90%)	3320 (97.41%)	
Emphysema, n (%)					< 0.001
YES	176 (1.62%)	37 (0.80%)	29 (1.01%)	110 (3.22%)	
NO	10,666 (98.37%)	4551 (99.19%)	2817 (98.98%)	3298 (96.77%)	
Chronic bronchitis, n (%)					< 0.001
YES	554 (5.10%)	155 (3.37%)	135 (4.74%)	264 (7.74%)	
NO	10,288 (94.89%)	4433 (96.62%)	2711 (95.25%)	3144 (92.25%)	
Cancer, n (%)					< 0.001
YES	891 (8.21%)	492 (10.72%)	207 (7.27%)	192 (5.63%)	
NO	9951 (91.78%)	4096 (89.27%)	2639 (92.72%)	3216 (94.36%)	
Depression, n(%)					
YES	9978 (92.03%)	4383 (95.53%)	2620 (92.05%)	2975 (87.29%)	
NO	864 (7.96%)	205 (4.46%)	226 (7.94%)	433 (12.70%)	< 0.001

##### Association between serum cotinine levels and depression

After adjusting for multiple confounders, Model II confirmed that there was a positive association between serum cotinine levels and the risk of developing depression. Specifically, for every 0.01 ng/mL increase in the serum cotinine concentration, the likelihood of developing depression increased by 0.14% (OR = 1.0014, 95% CI: 1.0009–1.0019, *p* < 0.001). This association was also observed when converting continuous variables to categorical variables. Compared to that in the T1 group, the risk of developing depression increased as the serum cotinine level increased in both the T2 group (OR = 1.45) and the T3 group (OR = 2.13) (Table 9).

**Table 9:** Weighted multivariable-adjusted logistic regression of Serum cotinine levels and depression risk

Exposure	Non-adjusted model	Adjust moedel I	Adjust model II
Serum cotinine levels category			
<=0.04	1	1	1
> 0.04, <=2.99	1.84 (1.51, 2.24) < 0.001	1.58 (1.29, 1.94) < 0.001	1.45 (1.17, 1.78) < 0.001
> 2.99	3.11 (2.61, 3.69) < 0.001	2.36 (1.94, 2.87) < 0.001	2.12 (1.73, 2.60) < 0.001
Serum cotinine levels Continuous	1.0021 (1.0017, 1.0025) < 0.001	1.0014 (1.0010, 1.0019) < 0.001	1.0014 (1.0009, 1.0019) < 0.001
P for trend	*P*<0.001	*P*<0.001	*P*<0.001

### Mendelian randomization study

#### MR results of smoking status and depression

**Fig. 5:**
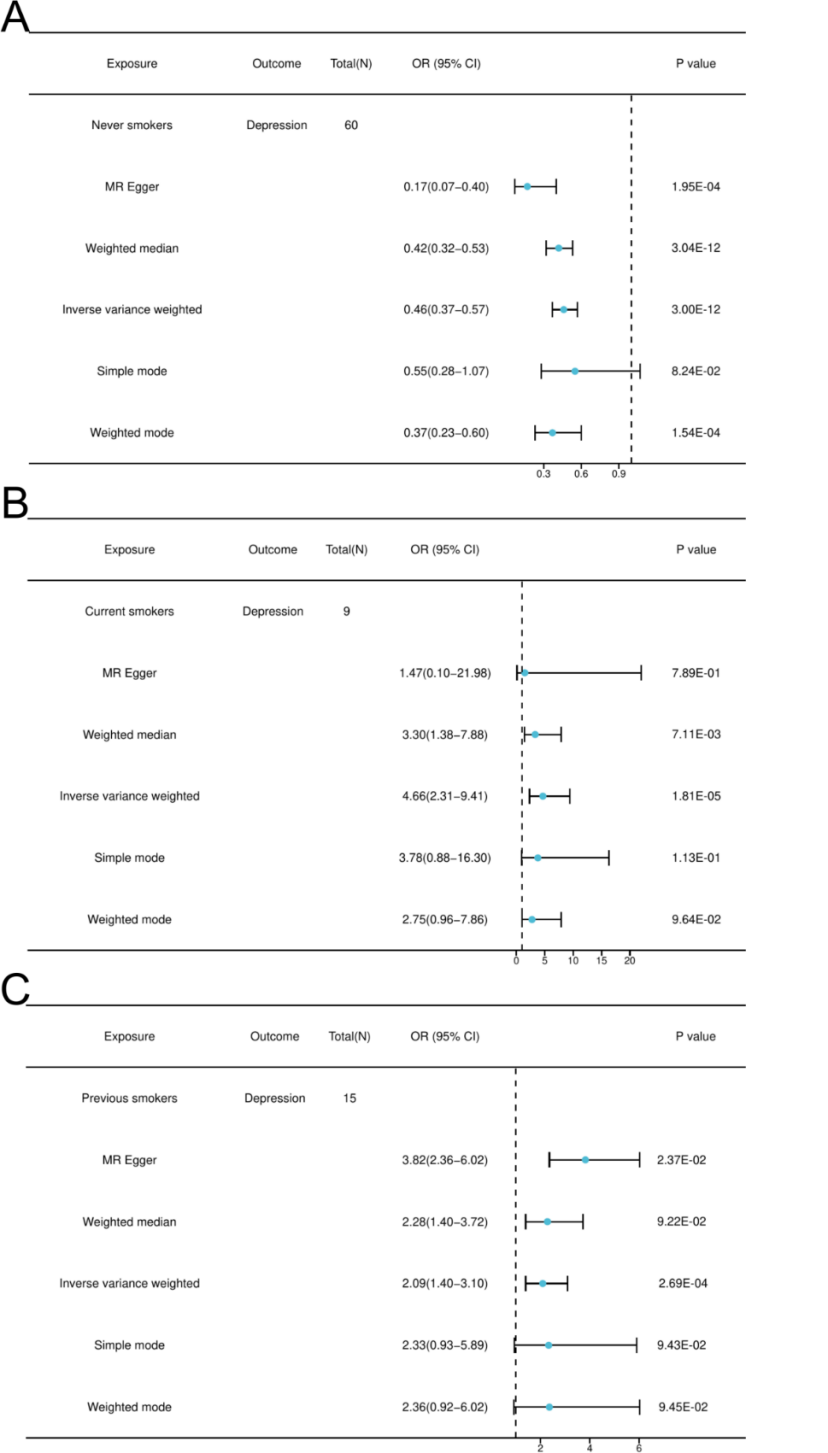
MR Result. (**A**) Never smoker (**B**) Current smoker (**C**) Previous smoker

Smoking status was classified as never smoked, previously smoked, or currently smoking. Eligible SNPs were screened from each of the three UKB datasets, and only SNPs with F-statistics greater than 10 (Table S6) were included to avoid bias from weak instrumental variables. The MR results showed (Fig. 5A-C) that in the IVW model, never smoking, past smoking, and current smoking were causally associated with the risk of developing depression, with the former being a protective factor and the latter two being risk factors. Current smokers had a 2.57-fold greater risk of developing depression than did previous smokers.

MR–Egger and IVW were used to test the heterogeneity of the MR results, and the data showed that some MR results were heterogeneous (*P* < 0.05). However, this did not affect the reliability of the MR results (Table 10). MR–Egger and MR–PRESSO were used to test the multivariate validity of the MR results, and the P–value for both methods was > 0.05, indicating that there was no horizontal pleiotropy in the MR results (Table 11).

**Table 10:** Heterogeneity cochran Q test result

Exposure	Outcome	Method	Cochran Q	*P*
Never smoker	Depression	Inverse variance weighted	107.38	0.30
		MR Egger	117.91	0.12
Current smoker	Depression	Inverse variance weighted	10.72	0.15
		MR Egger	11.87	0.57
Previous smoker	Depression	Inverse variance weighted	15.58	0.27
		MR Egger	20.69	0.11

**Table 11:** Test results for genetic pleiotropy

Exposure	Outcome	Method	Intercept b	*P* value
Never smoker	Depression	MR Egger	0.03	0.13
		MR Presso	-0.72	0.44
Current smoker	Depression	MR Egger	0.01	0.41
		MR Presso	0.15	0.20
Previous smoker	Depression	MR Egger	-0.02	0.06
		MR Presso	0.06	0.13

The funnel plot indicates that the SNPs that met the screening criteria were mostly symmetrical and did not exhibit significant outliers, thus increasing the robustness of the MR results. To test the sensitivity of the IVW results, we used the leave-one-out method. After removing any one SNP, the results of the remaining SNPs were on the same side of the null line, indicating that the removal of any one SNP did not significantly affect the results. Forest plots for individual SNPs enhanced the confidence of the MR results. Scatter plots demonstrating the correlation trend between exposure and outcome (Figure S1-S3).

#### MR results of cotinine levels and depression

Eligible SNPs were screened from the EBI dataset, and SNPs with F-statistics < 10 were excluded to avoid bias from weak IVs (Table S6). The MR results (Figure S4 and Table S7) showed no significant causal relationship between cotinine levels and the risk of developing depression in the IVW model (OR = 0.99, 95% CI: 0.97–1.02, *p* = 0.63).

The MR results were tested for heterogeneity and horizontal pleiotropy. The results indicated no significant heterogeneity or horizontal pleiotropy (Table S8 and S9). Additionally, the reliability of the MR results was further validated using funnel plots, leave-one-out plots, forest plots, and scatter plots (Figure S5).

## Discussion

This study collected data from the NHANES database (2005–2018) to analyze the correlation between TSE and depression from two perspectives: smoking behavior (including smoking status, smoking volume, and smoking cessation duration) and serum cotinine levels. It is the first study to combine large-scale genetic data with a large sample observational study to analyze the relationship between TSE and depression.

analyzedFrom the perspective of smoking status, smokers were found to have a significantly higher likelihood of depression compared to non-smokers, with current smokers having the highest risk. This supports the view that smoking increases the risk of depression [34]. In terms of smoking amount, a higher daily smoking amount was associated with a greater risk of depression, indicating a positive correlation between smoking amount and depression risk, which is consistent with previous research [35]. These findings suggest that even after adjusting for other covariates, the relationship between smoking and increased risk of depression remains, further supporting the conclusion that smoking increases the risk of depression. MR results indicate that both former smokers and current smokers increase the risk of depression, acting as risk factors for depression. The consistency between MR analysis and observational studies enhances the credibility of this finding.

Previous research has shown a positive association between smoking status and the risk of developing mental disorders such as depression, anxiety, and schizophrenia, with the smoking rate increasing with the severity of these diseases. Compared to nonmental disorder patients, individuals with mental disorders tend to start smoking at an earlier age, and they generally smoke more [36, 37]. Some scholars believe that smoking-induced anxiety or depression is mediated by its impact on individual neural circuits, increasing susceptibility to environmental stressors. In animal models, long-term nicotine exposure disrupts the hypothalamic‒pituitary‒adrenal axis, leading to excessive cortisol secretion, and alters the activity of relevant monoamine neurotransmitter systems, which regulate responses to stressors, and these effects seem to normalize after nicotine withdrawal [38]. In the analysis of the association between smoking status and the risk of developing depression, this study revealed that although the risk of developing depression is lower in former smokers than in current smokers, their risk of developing depression is still greater than that of those who have never been exposed to nicotine, which are possibly due to the long-term neurotoxic effects of nicotine [39]. While the short-term pharmacological effects of nicotine involve brain stimulation and relief from stress, anxiety, and depression, long-term nicotine use can lead to addiction, mental and psychological harm, increased sensitivity and vulnerability, and increased susceptibility to developing depression or anxiety [40].

analyzedanalyzedSerum cotinine levels have been used as a biomarker for exposure to tobacco smoke, so serum cotinine levels can reflect the current smoking status of participants to some extent. We divided serum cotinine levels into three groups based on previous studies, with the T3 group (> 3 ng/ml) representing serum cotinine levels in smokers. Both the unadjusted model and adjusted model, revealed that higher serum cotinine levels were associated with a greater likelihood of depression, and smokers (T3 group) had a significantly greater risk of developing depression than nonsmokers non-smokers (T1 group and T2 group). However, the MR results indicated no causal relationship between cotinine levels and depression risk. In recent years, in-depth research on cotinine has been conducted, and this compound has been applied in many other studies, including its use in treating various diseases.

However, research results on the effects of cotinine exposure are still varied. A cross-sectional study revealed that exposure to environmental tobacco smoke resulting in high serum cotinine levels significantly increased the risk of systemic inflammation and depressive symptoms. The study also revealed a linear dose‒response relationship between serum cotinine levels and the risk of depressive symptoms, which is consistent with the results of our observational study [41]. However, studies have shown that cotinine, a known antidepressant, can reduce cognitive deficits associated with disease- and stress-induced dysfunction. Its mechanism of action is mediated by the Akt/GSK3/synuclein pathway. Additionally, its pharmacokinetics suggest that cotinine has a favorable safety profile and is non‒addictive [42, 43]. This contradicts our results. Compared to observational studies, MR analyses provide more in-depth evidence but still cannot establish a causal link between cotinine levels and the risk of developing depression.

Our study’s greatest strength lies in combining a large sample observational study based on the NHANES with two-sample bidirectional MR analysis. This observational study allows us to explore the relationship between TSE and depression risk from an epidemiological perspective, while MR analysis addresses the inherent effects of residual confounding, reverse causality, and measurement errors in traditional epidemiological investigations. Although MR has a false-negative rate, the consistency between the two methods enhances the credibility of the study’s conclusions.

There are also limitations in this study. First, when conducting the cross-sectional study analysis, we excluded participants with incomplete data, which may introduce selection bias. Second, the PHQ-9 used in the NHANES to assess depression is an effective screening tool for assessing the frequency of depressive symptoms but is not a diagnostic tool for clinical depression. Last, our cross-sectional study and genetic data are not from samples of the same ethnicity, and the results are limited to Europeans and Americans. Therefore, these findings cannot be extrapolated to other populations, and future studies need to include individuals with the same ethnicity to eliminate potential confounding variables due to population heterogeneity.

## Conclusion

In summary, the findings indicate a strong association between smoking and depression, with smoking being a risk factor for depression development. Smoking cessation, on the other hand, reduces the risk of depression, and the longer the cessation duration, the lower the risk of depression. Observational studies have shown a significant association between cotinine and depression. However, MR results did not establish a causal relationship between cotinine and depression. Further validation is required through larger prospective cohort studies with a more rational design to explore the underlying mechanisms.

### Electronic supplementary material

Below is the link to the electronic supplementary material.

Supplementary Material 1

Supplementary Material 2

## Data Availability

All data in the manuscript are based on public data for secondary data analysis without ethical approval or primary data, and all data can be accessed at the following websites NHANES database: (https://wwwn.cdc.gov/nchs/nhanes/) IEU database: (https://gwas.mrcieu.ac.uk/) UKB database: (https://www.ukbiobank.ac.uk) EBI database: (https://www.ebi.ac.uk)
