# Supplementary material for: Association between tobacco smoke exposure and depression: the NHANES 2005–2018 and Mendelian randomization study
Source: Arch Public Health. 2024 Jul 3;82:100. doi: 10.1186/s13690-024-01322-4 (PMC11221044; doi:10.1186/s13690-024-01322-4)
Supplement: Supplementary file 1 — Supplementary Material 1 [file 13690_2024_1322_MOESM1_ESM.docx]

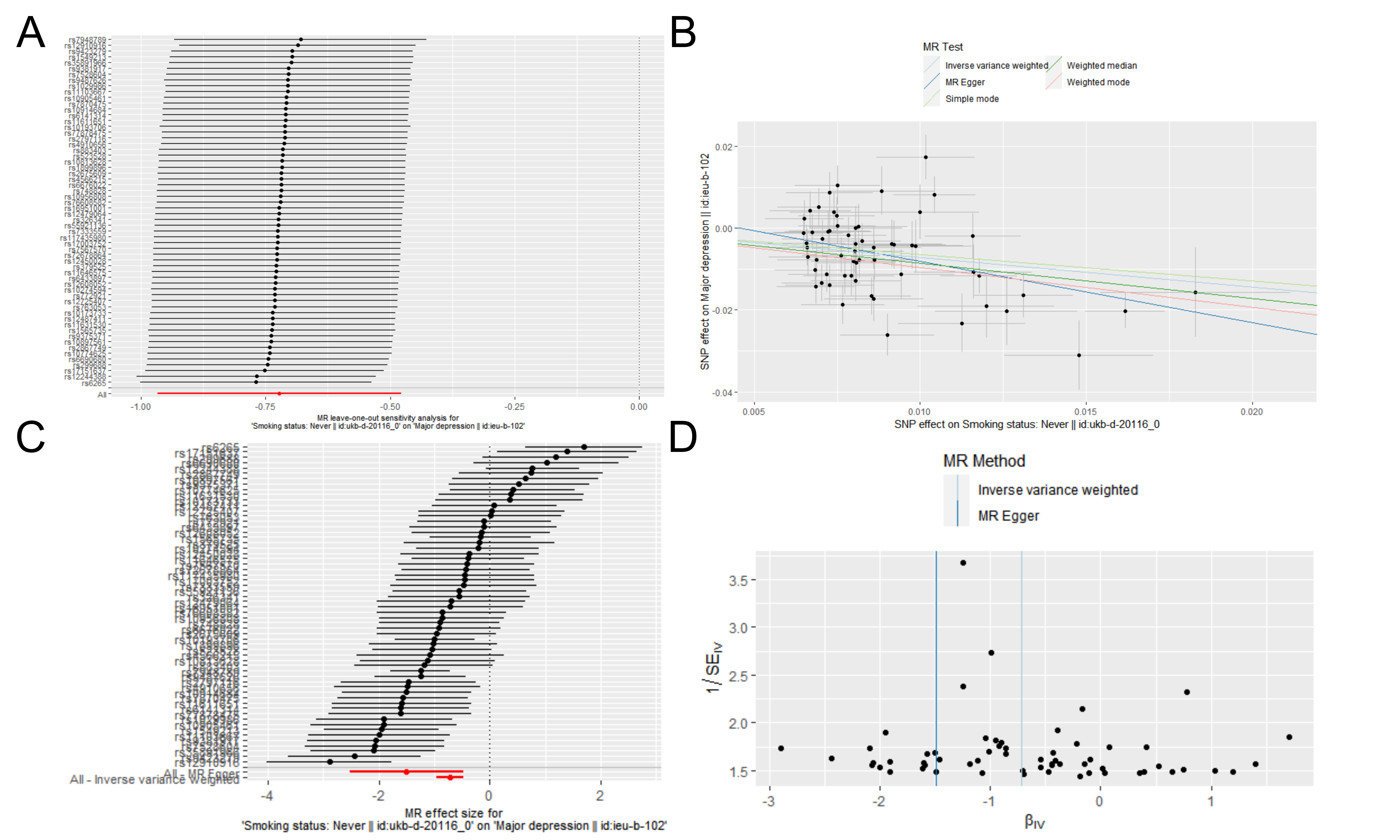


Figure S1. Never smoker (A) Leave-one-out plot. (B) Scatter plot. (C)Forest plot. (D) Funnel plot


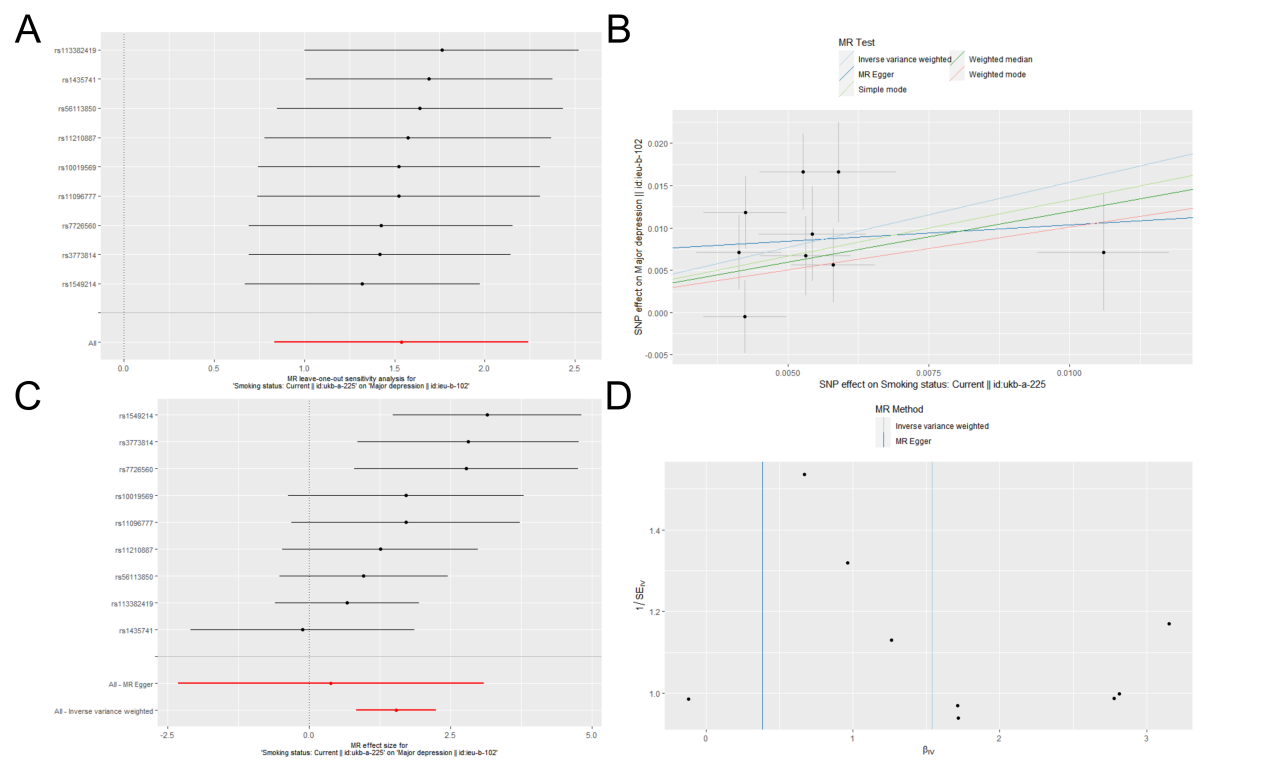


Figure S2. Current smoker (A) Leave-one-out plot. (B) Scatter plot. (C)Forest plot. (D) Funnel plot


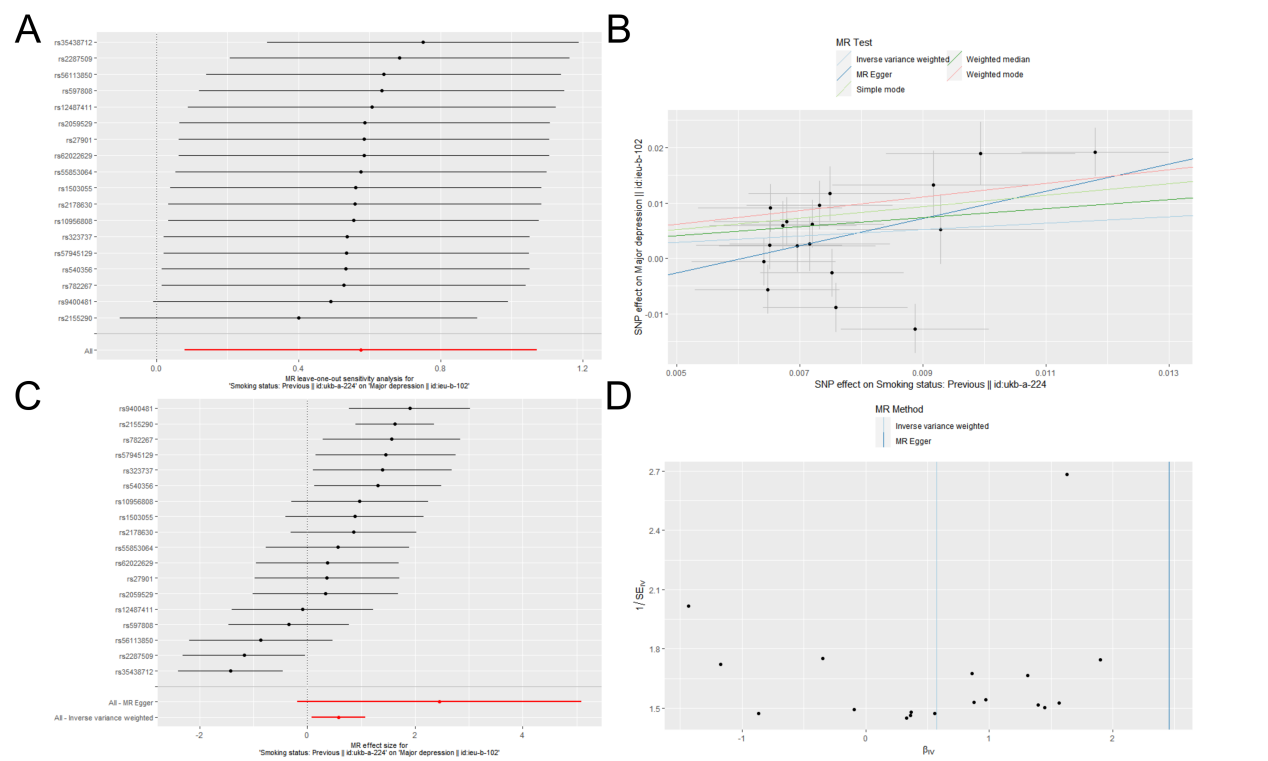


Figure S3. Previous smoker (A) Leave-one-out plot. (B) Scatter plot. (C)Forest plot. (D) Funnel plot


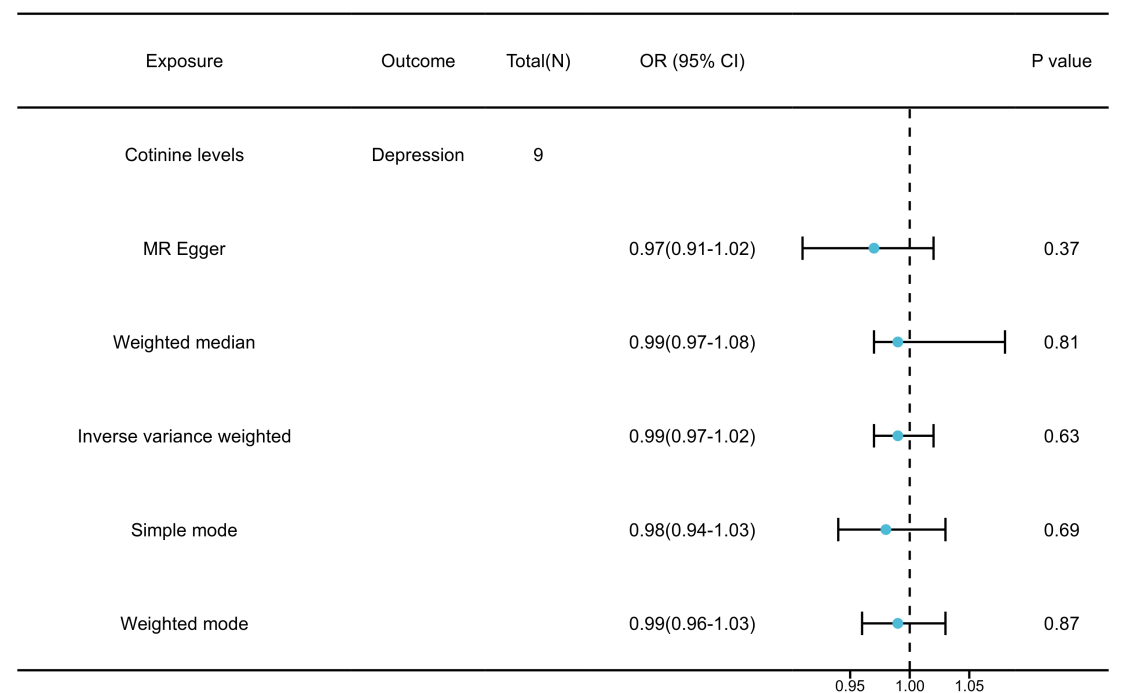


Figure S4. MR forest map


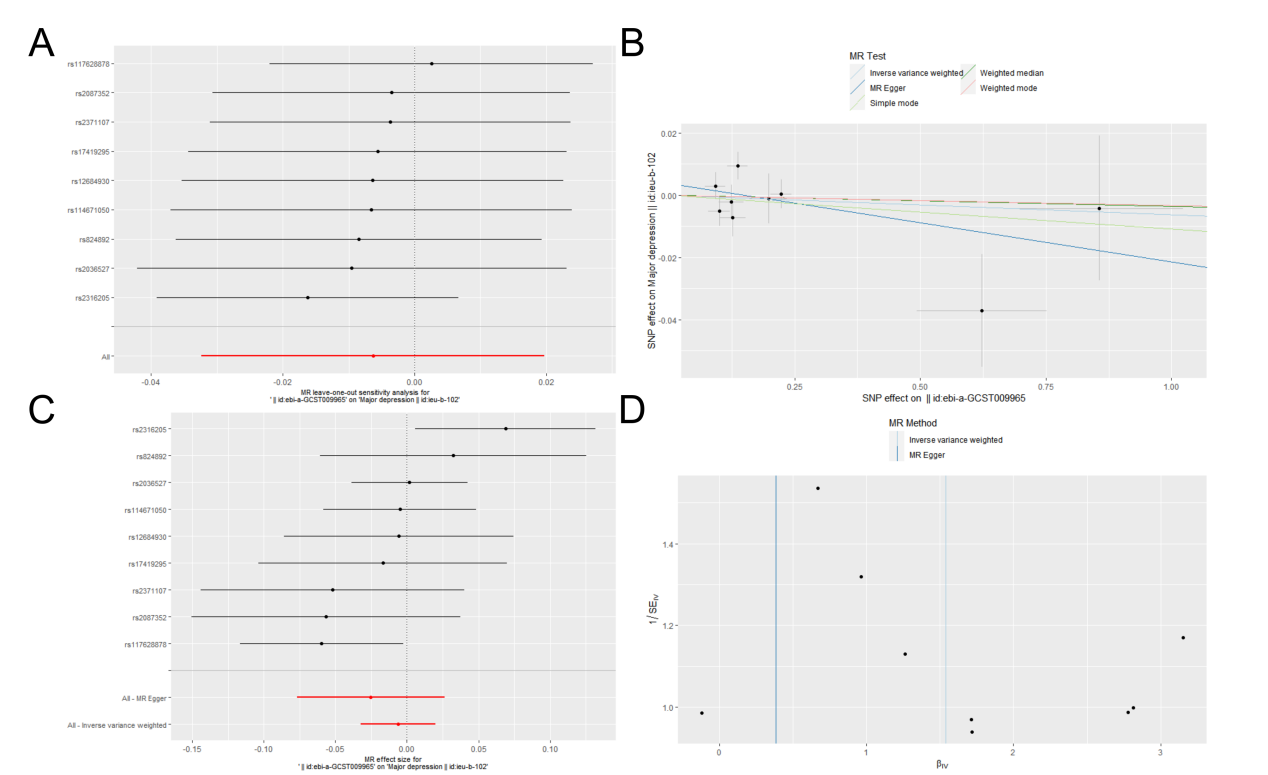


Figure S5. Cotinine level (A) Leave-one-out plot. (B) Scatter plot. (C)Forest plot. (D) Funnel plot
